# Multiscale Experimental Evaluation of Agarose-Based Semi-Interpenetrating Polymer Network Hydrogels as Materials with Tunable Rheological and Transport Performance

**DOI:** 10.3390/polym12112561

**Published:** 2020-10-31

**Authors:** Monika Trudicova, Jiri Smilek, Michal Kalina, Marcela Smilkova, Katerina Adamkova, Kamila Hrubanova, Vladislav Krzyzanek, Petr Sedlacek

**Affiliations:** 1 Faculty of Chemistry, Brno University of Technology, Purkynova 118, 61200 Brno, Czech Republic; xctrudicova@fch.vut.cz (M.T.); smilek@fch.vut.cz (J.S.); kalina-m@fch.vut.cz (M.K.); smilkova@fch.vut.cz (M.S.); 2 Institute of Scientific Instruments of the Czech Academy of Sciences, Kralovopolska 147, 61264 Brno, Czech Republic; katka@isibrno.cz (K.A.); hrubanova@isibrno.cz (K.H.); krzyzanek@isibrno.cz (V.K.)

**Keywords:** hydrogels, semi-interpenetrating polymer networks, controlled release systems, rheology, diffusion, cryo-scanning electron microscopy

## Abstract

This study introduces an original concept in the development of hydrogel materials for controlled release of charged organic compounds based on semi-interpenetrating polymer networks composed by an inert gel-forming polymer component and interpenetrating linear polyelectrolyte with specific binding affinity towards the carried active compound. As it is experimentally illustrated on the prototype hydrogels prepared from agarose interpenetrated by poly(styrene sulfonate) (PSS) and alginate (ALG), respectively, the main benefit brought by this concept is represented by the ability to tune the mechanical and transport performance of the material independently via manipulating the relative content of the two structural components. A unique analytical methodology is proposed to provide complex insight into composition–structure–performance relationships in the hydrogel material combining methods of analysis on the macroscopic scale, but also in the specific microcosms of the gel network. Rheological analysis has confirmed that the complex modulus of the gels can be adjusted in a wide range by the gelling component (agarose) with negligible effect of the interpenetrating component (PSS or ALG). On the other hand, the content of PSS as low as 0.01 wt.% of the gel resulted in a more than 10-fold decrease of diffusivity of model-charged organic solute (Rhodamine 6G).

## 1. Introduction

Hydrogel represents a three-dimensional, water-swollen network assembled from cross-linked chains of either polymer molecules or partially coagulated colloidal particles. Because a macromolecular gel network can be formed from virtually any water-soluble polymer, hydrogel materials generally encompass a wide range of chemical compositions possessing a great variety of miscellaneous physicochemical functionalities that can be exploited in numerous applications [1]. Among them, the biomedical use of hydrogels has attracted particular interest since their first appearance. In a fact, hydrogels were the first biomaterials designed intentionally for the use in the human body [2,3]. The main benefits regarding their biomedical application include a combination of high water content and physicochemical similarity to the native extracellular matrix [4] which together results in their high biocompatibility. Furthermore, their internal structure, as well as the rate of degradation or dissolution in vivo, can be tuned by controlling their chemical composition, type and density of the cross-links, etc. Since the first pioneer works on covalently cross-linked poly(2-hydroxyethyl methacrylate) gels published by Wichterle and Lim in 1960 [3], significant progress has been made in the field of hydrogel design for biomaterial use, in particular in improving the physical form of the hydrogel delivery (including micro- or nanoparticulate gels [5,6], gel films [7,8], etc.), in providing a specific response to a change in the external conditions (such as temperature [9,10], pH [11,12] or concentration of a particular biomolecule [13,14]), or in obtaining materials with precisely designed internal architecture such as the superporous gels [15,16], hydrogels self-assembled from biopolymers produced by genetically engineered microorganisms [17,18], dual network gels [19,20] and many others. Hence, until now, hydrogel materials have not only gained a prominent position in the field of medical research but have also been put into common practice in tissue engineering [21,22], regenerative medicine [23,24], diagnostic [25,26] or separation techniques [27,28], cell immobilization and cultivation and, perhaps most exclusively, in drug-delivery applications [29].

Despite many beneficial properties, hydrogels have also some specific limitations and risks regarding their uses in the development of drug-delivery systems [29]. For example, poor mechanical properties (i.e., low tensile strength) of the gels compared to ‘hard’ drug-delivery systems such as nanoparticles may lead to a premature disintegration of the gel carrier and instantaneous release of the active substance under a mechanical strain [30]. Perhaps even more important are the specific limitations connected to the transport properties of the gels. Drug loading capacity, as a complex product of multiple influences (drug solubility in water, its partition between gel and solution, or its effect on the stability of the gel network junctions) is usually limited even in the case of hydrophilic active substances [31,32]. Moreover, high water content and large pore size indispensably result in rapid drug release on a time scale of hours to days which again barely competes with the long-term release profiles of other delivery systems such as microspheres [33]. Until now, a range of strategies has been proposed to improve the partition of the active substance in the gel and to retard its release from the hydrogel carrier. These strategies usually aim at supporting the binding (either chemical or physical) between the drug and the polymer network. Hence, numerous procedures have been explored to incorporate monomers with a specific ionic or non-ionic affinity to the drug into the preparation of synthetic polymer networks [34], to exploit the presence of native functional groups of biopolymers (e.g., carbohydrates) in their physically cross-linked gels [35,36] or to conjugate the drug to the gel network via covalent bonds prone to enzymatic or chemical cleavage in situ [37].

In the present work, we put forward an alternative strategy, which aims to address both the aforementioned issues of the hydrogel drug carriers independently to each other. Our hydrogel system is based on semi-interpenetrating polymer networks (semiIPNs) which, together with interpenetrating polymer networks (IPNs), belong to the class of polymer blends. IPNs are defined by International Union of Pure and Applied Chemistry (IUPAC) as “A polymer comprising two or more networks which are at least partially interlaced on a molecular scale but not covalently bonded to each other and cannot be separated unless chemical bonds are broken.” [38] SemiIPNs differ from the IPNs in the fact that the chains of the second polymer are dispersed in the network formed by the first polymer without forming a separate network and, consequently, the linear polymer component can in principle be separated from the constituent polymer network without breaking chemical bonds [38]. In recent years, both IPNs and semiIPNs have attracted special attention in novel material applications mainly due to the possibility of combining favorable properties of each polymer component to the final properties superior to those provided by the two polymer constituents alone. Hence, several IPN- and semiIPN-based hydrogel compositions have recently been proposed for application in tissue engineering [39] and in controlled-release (CR) systems [40], where the synergistic effect brought by the combination of the two polymer component is primarily focused on the specific improvement of mechanical properties, biocompatibility, thermal stability or chemical resistance of the gel.

In our study, we introduce a different approach to the utilization of semiIPN hydrogels in drug-delivery systems. Rather than focusing on a specific polymer composition developing carriers of hydrophilic (in particular ionic) active compounds based on semiIPN hydrogels which comprise a ‘structure–ruling’ gel-forming component, with a low affinity to the drug substance, interpenetrated by a ‘binding’ component that does not interfere with the internal morphology and the mechanical properties of the gel but significantly improves hydrogel reactivity. We hypothesize that such a system would allow an independent dual-tuning of mechanical and transport performance via manipulating the relative content of the two structural components. As a prototype material, we here introduce hydrogels based on an agarose network interpenetrated by a linear polyelectrolyte component. Agarose is involved in the study as a model representative of thermomelting polysaccharides which form physical hydrogels via thermally induced phase separation. Among versatile applications of agarose [41], its use in tissue engineering [42] and drug delivery [43] has recently attracted special attention mainly for its great biocompatibility, temperature-dependent behavior [43,44], and multiple options of manipulating its internal architecture [45,46]. As an interpenetrating component, two structurally distinct linear polyanions, alginate and poly(styrenesulfonate), are used in this study to provide an attractive binding affinity towards model low-molecular solute—Rhodamine 6G. This compound was used mainly for its complex molecular structure combining highly hydrophilic positively charged nitrogen atom with neighboring aromatic structural moieties, which represents a common structural motif widespread in different groups in pharmaceuticals [47,48].

The main aim of the study is to test the ability of the system to tailor the chemical structure and internal morphology of the proposed semiIPN hydrogels separately and, consequently, to manipulate its mechanical (rheological) and transport properties independently. To evaluate the relation between internal structure (both physical and chemical), mechanical properties, and transport performance of the resulting gels, we present a unique analytical approach, where the three fundamental material qualities (structure, rheological properties, and transport performance) are studied both on the macroscopic (i.e., the sample-averaged) and the microscopic scale.

## 2. Materials and Methods

### 2.1. Preparation of the Gels

All hydrogels, utilized in this study were prepared via the thermoreversible gelation of the aqueous solution of agarose (Type I, low electroendoosmosis; Sigma-Aldrich, Prague, Czech Republic). Agarose hydrogels (without any addition of a polyelectrolyte component) were prepared from the aqueous solution of agarose (concentration of agarose in solution/gel: 0.5%, 1%, 2%, and 4% by weight), while agarose-based semiIPN gels from the aqueous solution of agarose (1 wt.%) with a corresponding addition of a dissolved polyelectrolyte (0.002, 0.005 and 0.010% by weight). As a polyelectrolyte component, alginic acid sodium salt (ALG; Sigma-Aldrich, Prague, Czech Republic, 180947, average MW 120–160 kDa, M/G ratio 1.33 [49]) and poly(sodium 4-styrenesulfonate) (PSS; Sigma-Aldrich, Prague, Czech Republic, average MW 70 kDa), respectively, were used.

The gelation proceeded as follows: the accurately weighed amount of agarose powder was dispersed in deionized water (preparation of agarose gels) or in the aqueous solution of the respective polyelectrolyte of the required concentration (semiIPN gels), respectively. The mixture was at first slowly heated with continuous stirring to 85 °C and then maintained at the constant temperature until the solution turned transparent. Subsequently, the solution was degassed in an ultrasonic bath (1 min. at 85 °C) and slowly poured into the corresponding container (according to the needs of subsequent analysis) which was then stored in a closed bottle above the water-level (i.e., at 100% relative humidity to prevent unwanted surface evaporation of water). Upon the gradual cooling to room temperature (approximately 45 min), the mixture gradually gelled.

### 2.2. Turbidimetry

For the turbidimetric experiment, the respective heated and degassed agarose solution (with or without the respective polyelectrolyte component) was poured and let to cross-link in the poly(methyl methacrylate) cuvettes for spectrophotometry (10 × 10 × 45 mm^3^). Subsequently, the ultraviolet–visible (UV-VIS) transmittance spectrum of the gel was collected in the wavelength range 300–800 nm on Hitachi U3900 spectrophotometer (Tokyo, Japan), whereby deionized water was used as a reference sample. From the transmittance spectrum, optical densities (OD) in the spectral range of 700 to 800 nm (where no specific light absorption by the gel components is expected) were calculated according to OD (λ) = −log*T*(λ). From the optical density, turbidity was calculated (considering optical path length = 10 mm) from τ(λ) = 2.3 OD (λ). Calculated turbidities in the spectral range 700–800 nm were plotted as logτ(λ) = *f*(logλ) and the linear fit of the plot was performed using MS Excel. The wavelength exponent then was determined as the slope of the linear fit and further transformed into the correlation length (in µm) using data published by Aymard [50]. Finally, the correlation length value is presented as an effective value of mesh size determined from turbidimetry.

### 2.3. Oscillatory Rheometry

For the analysis of macroscopic viscoelastic behavior of a hydrogel sample, the circular cut of the gel (40 mm in diameter, 1.1 mm in height), gelled in a Petri dish before the analysis, was placed on the bottom Peltier plate of the Rheometer AR-G2 (TA Instruments, New Castle, DE, USA) pre-tempered to 25 °C. The oscillatory analysis was performed using plate-plate geometry (titanium plate sensor, 40 mm in diameter) at a constant temperature of 25 °C. The upper rheometer shaft with geometry was moved into the trim gap (1020 μm) and excess of hydrogel outside of both plates was cut away by a spatula. The geometry gap (1000 μm) was reached (normal force during compressing did not exceed 5 N). Due to the high content of water in hydrogels, the solvent trap was used to prevent the potential change in viscoelastic properties due to the evaporation of the dispersion medium. Frequency sweep measurements were performed on the whole set of hydrogel samples in duplicate. The conditioning step (25 °C, 5 min) preceded before each measurement. Thanks to the conditioning step, each hydrogel was relaxed and tempered to the required temperature before measurement. Firstly, the linear viscoelastic region (LVR) was determined by strain sweep test at a constant frequency of oscillation (1 Hz) in the range 0.01–1000%, 6 points per decade (Figure S2 in Supplementary Materials). The constant amplitude of deformation chosen from the LVR (0.5%) was used for all frequency sweep measurements. Frequency sweep measurements were performed on the whole set of hydrogel samples in duplicate (at least) with parameters as follows: 0.01–20 Hz, 6 points per decade, decimal logarithmic mode. The relative deviation of the duplicate measurements never exceeded 10% (in terms of elastic (storage) modulus G’ and viscous (loss) modulus G’’, respectively). From the recorded values of G’ and G’’, the corresponding values of complex modulus $\left|G^{*}\right|\;$ and phase angle δ were calculated according to:

(1) $$\left|G^{*}\right|=\sqrt{{\left(G^{\prime }\right)}^{2}+{\left(G^{\prime \prime }\right)}^{2}}$$

(2) $$\delta =\mathrm{arctg}\frac{\;G\;\prime \prime }{\;G\;\prime }$$

To calculate the effective mesh size from frequency sweep data, frequency dependencies of elastic and viscous moduli were fitted (using least square regression algorithm) with a generalized Maxwell model [51] using the procedure described by Pescolido et al. [52]. Fitting functions were as follows:

(3) $$G^{\prime }=\sum_{i=1}^{n}G_{i}\frac{{\left(2\pi f\lambda_{i}\right)}^{2}}{1+{\left(2\pi f\lambda_{i}\right)}^{2}}$$

(4) $$G^{\prime \prime }=\sum_{i=1}^{n}G_{i}\frac{2\pi f\lambda_{i}}{1+{\left(2\pi f\lambda_{i}\right)}^{2}}$$

where *f* is the frequency of oscillations (in Hz), *n* = 4 is the number of considered Maxwell elements (determined via the statistical procedure described in [53]), $G_{i}$ and $\lambda_{i}$ represents the corresponding spring constant and relaxation time, respectively, of the *i*-th Maxwell element. Data fitting was performed using the Solver tool in MS Excel. Based on the results of the data fitting, hydrogel shear modulus was calculated as the sum of the spring constants of Maxwell elements $G=\sum_{i=1}^{n}G_{i}$ and transformed into the density of crosslinking ρ_x_ and into the average network mesh size ξ according to:

(5) $$\rho_{x}=\frac{G}{RT}$$

and,

(6) $$\xi =\sqrt[{3}]{\frac{6}{\pi \rho_{x}N_{A}}}\;$$

where *T* is the thermodynamic temperature, while *R* and *N_A_* the universal gas constant and Avogadro constant, respectively.

### 2.4. Microrheometry

A colloidal analyzer Zetasizer Nano ZS (Malvern Panalytical Ltd., Great Malvern, UK) was used to collect dynamic light scattering (DLS) microrheological data. This method is based on observing the movement of tracer particles with defined particle size (polystyrene monodisperse with nominal particle size 100 nm, Sigma-Aldrich, Prague, Czech Republic) in the sample via monitoring the time development of intensity of the light scattered by these particles. The tracer particles were homogeneously incorporated inside the analyzed hydrogels during the initial preparation step (they were added to the mixture before heating up to dissolve agarose powder). The experimental parameters of DLS microrheological measurements were set as follows: temperature: 25 °C, equilibration time: 60 s, duration of one run: 10 s, number of runs: 12, number of measurements of each sample: 5. Each sample was prepared and analyzed in three replicates. The main experimental outcomes from DLS microrheology was the dependence of mean square displacement (MSD) of tracer particles in the studied hydrogels on the observation time. From the respective MSD, viscoelastic parameters (primarily the storage and loss moduli) were calculated in the Microrheology software tool in Zetasizer Software (Malvern Panalytical Ltd., Great Malvern, UK). Other viscoelastic parameters (complex modulus, phase angle) were calculated using equations shown above for the oscillatory rheology.

### 2.5. Macroscopic Diffusion Experiments

The whole set of prepared hydrogels was subjected to diffusion experiments proposed and optimized in our previous study [54,55]. For this purpose, hydrogel samples were prepared directly in PMMA cuvettes for spectrophotometry similarly to turbidimetry analysis. In this case, the cuvettes were overfilled with the solution to achieve the concave meniscus of the agarose solution at the cuvette edge. After the solidification (approximately 30 min, room temperature), the excess hydrogel was cut away to obtain a flat hydrogel surface at the open orifice of the cuvette.

The hydrogel-filled cuvettes were then immersed in a solution of model solute to study its diffusion in the gels qualitatively and quantitatively. As the model solute, positively charged organic dye Rhodamine 6G (R6G, dye content > 95 wt.%, Sigma-Aldrich, Prague, Czech Republic) was used. The concentration of the source solution of R6G in the diffusion experiment was 0.01 g.dm^–3^, the solution was continuously stirred via a magnetic stirrer (250 RPM) during the whole diffusion experiment. At selected times (24, 48, and 72 h), the cuvettes were taken out of the solution and the UV-VIS absorption spectra were measured in the 300 to 800 nm spectral range at various distances from the orifice on Varian Cary 50 UV–VIS spectrophotometer (Varian, Inc., Paolo Alto, California, USA) equipped with the custom-made accessory providing controlled fine vertical movement of the cuvette in the spectrophotometer (for details on the accessory, see [56]). For a determination of R6G concentration in the gel from the recorded spectra, a set of reference hydrogel samples was prepared for every tested agarose-polyelectrolyte composition. In the reference hydrogel samples, a known concentration of homogeneously dispersed R6G was provided by the addition of the corresponding amount of R6G to the agarose solution before its gelation. Further data processing of the spectra (suppression of the light scattering background signal) was described in detail previously [54,56]. Diffusion experiments were performed in duplicates for each of the analyzed hydrogel compositions.

Each concentration profile (dependence of R6G concentration on the position in the gel, i.e., on distance from the solution/gel interface) determined for a particular hydrogel at the time of diffusion *t* were fitted by following diffusion equation (for the derivation, see [57]) using the Solver tool in MS Excel:

(7) $$c\left(x\right)=c_{0}\cdot \mathrm{erfc}\frac{x}{\sqrt{4D_{eff}t}}$$

where *c(x)* and *c_0_* are concentrations of R6G in the gel (in g·m^–3^) at distance *x* from the interface or at the interface, respectively, and *D_eff_* is the effective diffusion coefficient of R6G in the gel. From the fitting parameters (*D_eff_*, *c*_0_) the rate of R6G diffusion and its partition in the gel is described quantitatively. The partition coefficient is then calculated as the ratio of the interface concentrations of R6G in the gel and the solution ($c_{sol}$), respectively:

(8) $$\varepsilon =c_{0}/c_{sol}$$

Mean values and standard deviations of these parameters were calculated by averaging the results for three diffusion times and duplicated measurements.

### 2.6. Fluorescence Correlation Spectroscopy

Self-diffusion of the molecules of the model solute (R6G) was studied by fluorescence correlation spectroscopy (FCS). For this purpose, the homogeneous distribution of the R6G molecules (concentration in the order of nM) was achieved similarly to the preparation of reference hydrogel samples for the evaluation of macro-diffusion experiments.

The FCS measurements were performed on MicroTime 200 instrument (PicoQuant, Berlin, Germany) equipped with a fluorescence microscope Olympus IX71 (Olympus, Tokio, Japan) (setup of the system: laser wavelength 510 nm, dichroic mirror 514/640 nm, emission filter 550/49, laser intensity 6.6 μW). Moreover, during FCS measurements, two single photon avalanche diode detectors were used, which allowed us to use cross-correlation for data evaluation. To maintain uniform measurement conditions, at the beginning of the experiment the vertical (*xz*) scan was performed and the position of the glass-gel interface was identified. Afterward, a horizontal (*xy*) scan was performed 5 μm above the glass surface and three different positions were chosen for measurements for each sample. Subsequently, for FCS analysis each hydrogel sample was prepared in five replicates. The main outcome from FCS analysis is the coefficient of self-diffusion of R6G in each hydrogel sample.

### 2.7. Scanning Electron Microscopy Imaging

For the scanning electron microscopy (SEM) imaging of the internal structures of the gels, samples were first cryogenically fixed. Small copper-based thin-wall tubes with a diameter of approximately 1 mm were at first filled with the particular hydrogel sample—each tube was filled by performing a horizontal motion through the already gelled agarose or agarose/polyelectrolyte solution using tweezers so that the hydrogel protruded from the tube at both ends. The plunge-freezing technique of fixation was used, where a small amount of hydrogel is rapidly cooled by immersion in cryogen (liquid nitrogen in this case). After having been plunge-frozen, the tubes containing hydrogel samples were kept at cryogenic temperatures throughout the whole experiment including the imaging. Before the imaging, freeze-fracture was also applied by cutting off the protruding part and scratching the surface superficially with a sharp blade at high vacuum and low temperature in the EM ACE600 preparation chamber (Leica microsystems, Vienna, Austria). Three successive steps of freeze etching to reveal the internal microstructure were applied. During each of the etching steps, the temperature was increased to −100 °C, and after it stabilized, decreased back to −120 °C. All the frozen samples were imaged in the SEM Magellan 400L (FEI-Thermo Fisher Scientific, Hillsboro, OR, USA) equipped by a temperature-controlled cryo-stage at the temperature of −120 °C; the imaging was performed before any freeze etching and after each freeze etching step.

Image processing and analysis of the cryo-SEM images was performed in ImageJ open-source image processing toolbox (National Institute of Health, Bethesda, Maryland, USA and Laboratory for Optical and Computational Instrumentation, University of Wisconsin, Madison, USA; version 1.51, [58,59]). For this purpose, square sections of the raw cryo-SEM images (512 × 512 pixels) were processed by the following procedures: contrast and brightness of the section were first adjusted according to the image histogram and the bandpass filter was used to suppress lightening inhomogeneities and horizontal stripes. Then the processed images need to be converted from grayscale to black and white projections in an effort to display only the uppermost layer of the 3D network structure. For this conversion, the image sections were thresholded using the MaxEntropy algorithm. Subsequently, the actual analysis of the internal structure proceeded using ‘Analyze particles’ and ‘Analyze skeleton’ commands (details are provided in the Results section).

## 3. Results

### 3.1. Mechanical Properties of the Gels

**Figure 1:**
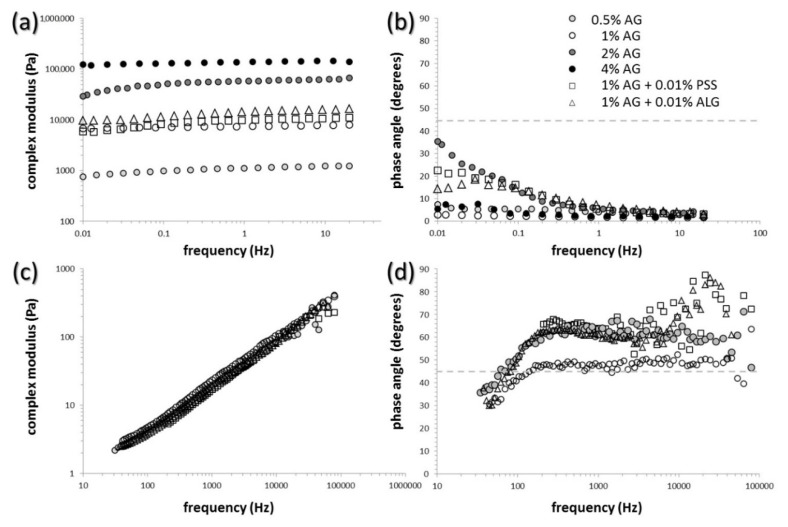
Parameters of viscoelasticity of the gels determined on macroscopic (**a**,**b**) and microscopic scale (**c**,**d**). Frequency dependence of the complex modulus (**a**,**c**) and the phase angle (**b**,**d**), respectively.

Overall viscoelastic properties of the semiIPN gel samples were examined by standard techniques of oscillatory rheometry. Results of the frequency sweeps are shown for agarose gels and selected semiIPN gels in Figure 1a,b (corresponding frequency dependencies of storage and loss moduli as the raw experimental data are provided in Figure S1 in Supplementary Materials). As can be seen, the value of complex modulus (calculated using Equation (1)) is almost frequency-independent for all analyzed hydrogels (see Figure 1a). This represents a characteristic rheological feature of densely cross-linked gel networks where the deformation response in the linear viscoelastic region is not significantly affected by the timescale of the deformation [60,61,62]. Perhaps more important, it can be seen in the same figure that the overall stiffness of semiIPN gels can be adjusted over a wide range of values by alternating the concentration of the network-making component. In the case of agarose gels, changing the concentration of agarose from 0.5% to 4% by weight shifts the complex modulus of the gels to the values higher by more than two orders of magnitude. On the other hand, the presence of the interpenetrating polyelectrolyte components (PSS and ALG) in the range of concentrations used in this work (max. 0.01% by weight of the gels) does not affect the stiffness of the gel severely (see Figure 1, for other concentrations of polyelectrolytes, see Figures S2 and S3 in Supplementary Materials). The frequency dependence of the phase-shift angle (calculated using Equation (2)) illustrates the relative contribution of the elastic and viscous type of deformation on different time scales (see Figure 1b). It can be seen that even at the lowest frequencies of the oscillatory shear deformation, the phase-shift angle is still lower than 45° for all prepared gels (including as well the semiIPN gels with lower content of polyelectrolyte component, data shown in Figure S3 Supplementary Materials). In other words, even for the slowest deformations that take place on the longest timescales, the gel is deformed predominantly elastically. At the deformation timescale of seconds, the value of phase angle is so low for all gels (<10°), that their deformation behavior resembles ideal solids. Finally, it should be also mentioned that all prepared hydrogels were subjected also to complementary oscillatory tests such as amplitude sweep (alternatively called strain sweep) and relaxation tests. Although all the particular results are not shown here (see results of the strain sweep test in Figure S4 in Supplementary Materials), they can be concluded similarly—differences in the deformation response of the gels caused by the presence of interpenetrating polyelectrolyte component was insignificant compared to the impact of the different content of the gel-forming component (agarose).

The microrheological assay describes the deformation behavior of the gels from a different perspective. The method based on DLS observation was used for monitoring the thermal motion of standard tracer microparticles incorporated into the internal structure of the gels. In this approach, from the scattering correlogram (a function which correlates the scattering intensity over time), the mean square displacement function of the tracer particles is derived and the basic viscoelastic parameters (storage and loss moduli and the others related with these two) are calculated for individual characteristic times or frequencies. This approach therefore provides a microrheometric analog to standard oscillatory frequency sweep tests—viscoelastic parameters which are obtained are the same in their essence for both methods; nevertheless, in the case of microrheology, they characterize viscoelasticity of that part of the internal space of the hydrogel where the thermal motion of the tracer particles takes place.

Basic results of the DLS microrheology are presented in Figure 1c,d and Figure S5 in Supplementary Materials. Figure 1c,d show frequency dependencies of complex moduli and phase angles, respectively, derived from the thermal motion of tracer microparticles in the internal pores of the particular hydrogel structure. As can be seen, the results of this experiment show fundamental differences as compared to the results of common oscillatory (macro)rheometry assay. Complex moduli of the gels determined by DLS microrheometry are significantly lower and considerably more dependent on the frequency of the deformation. Furthermore, as far as the frequency-dependent complex moduli (as well as complex viscosity, an alternative quantitative parameter describing stiffness of the material) of all tested gels (again, including the other PSS and ALG containing semiIPN gels presented graphically in Figure S5 in Supplementary Materials) show virtually the same values, and it can be deduced that the local environments of the microparticle motion in particular gels provide similar deformation response. Contrarily to the classical oscillatory rheometry, the viscous character of the deformation behavior predominates (note the values of phase angle > 45° in Figure 1d). This is reasonable from the point of view that the thermal motion of the particles takes place in the liquid pores of the hydrogel matrix and that the microrheological approach, therefore, provides insight into the deformation behavior of this local microenvironment. Furthermore, it can be seen from the frequency dependencies of phase angles that while the lowest applied frequencies induce the most viscous deformation response in the case of oscillatory macrorheometric analysis, the opposite is observed for the microrheology assay (most elastic response is measured for the lowest characteristic frequencies). Again, this apparent discrepancy of the viscoelastic properties arises from the essential difference between the two rheometric approaches. While for the oscillatory rheometry the lowest frequencies characterize the slowest applied oscillatory deformation where the liquid-like character of the material is most manifested, in the case of microrheology the lowest frequencies correspond to the longest correlation time where the trace particle motion reaches also more distant surroundings of the particle. Therefore, the presence of a polymer network that surrounds liquid pores filled with tracer particles affects the particle motion most strongly just for the lowest frequencies. It can be seen in Figure 1d that concerning both discussed aspects of the viscoelastic response of tested gels on the microscopic scale, no fundamental differences were found for the gels no matter what the content of gel-forming or interpenetrating component was.

### 3.2. Transport Properties of the Gels

**Figure 2:**
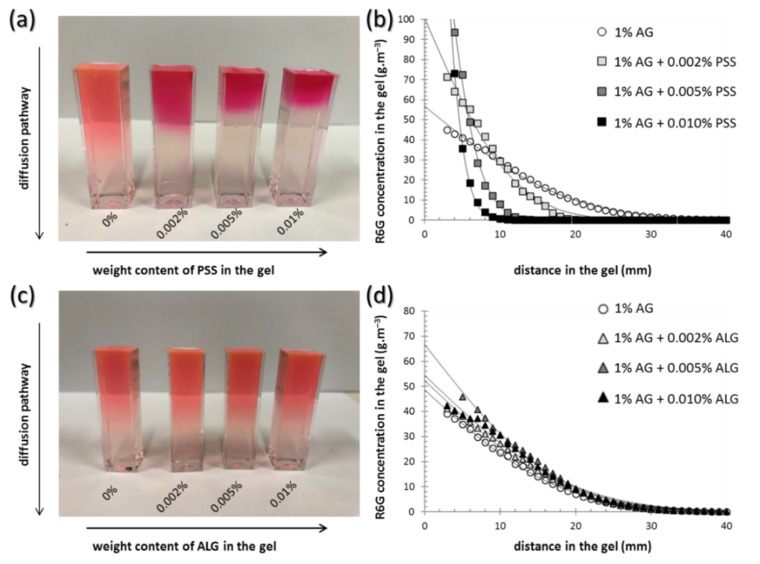
Diffusion of Rhodamine 6G (R6G) in the gels investigated on the macroscopic scale. (**a**,**c**) Visual comparison of agarose gels (1 wt.% of agarose) with different content of the interpenetrating component after 72 h of diffusion of R6G from solution. (**b**,**d**) Experimentally determined concentration profiles of the same gels.

Similarly to the investigation of mechanical properties of the gels, also the analysis of transport performance towards the model hydrophilic solute (Rhodamine 6G, R6G) was performed using both a macro- and a micro-scale approach. The results of the diffusion experiments are shown in Figure 2 and Table 1 and Table 2. Firstly, macroscopic investigation of the diffusion of R6G from source solution into the gels was involved using the methodology developed in our previous work [37,38,39]. It can be seen in Figure 2a,c that, using this simple method, the effects of the interpenetrated components on the rate of transport in the gels can be evaluated even visually. In particular, the picture in Figure 2a shows that the presence of interpenetrating PSS chains severely affects (decelerates) the rate of R6G diffusion in the gel and that the effect correlates with the content of PSS in the gels. Furthermore, different color saturation in the gel near the interface with the source solution indicates that also the partition of the R6G between the solution and the gel is affected by the presence of PSS. On the other hand, no such effects can be observed in the case of ALG’s presence in the gels (see Figure 2c). These visual observations can easily be transformed into quantitative information by measuring UV-VIS spectra at different positions in the gels. Using the calibration method based on gels prepared with a known content of R6G, concentration profiles of R6G can be determined for different gel compositions and for various times of the diffusion experiment. A comparison of such concentration profiles corresponding to gels with different contents of PSS and ALG is shown in Figure 2b,d, respectively.

By the regression of the concentration profiles (using fitting Equation (7)), effective diffusion coefficients (*D_eff_*) and boundary concentrations of R6G in the gel (*c_0_*) can be calculated (see Table 1 and Table 2). It can be seen that the comparison of absolute values of the calculated diffusivities agrees well with the qualitative features discussed based on the visual investigation of the gels. The presence of PSS in the gel reduces the effective diffusivity of R6G significantly; the highest content of PSS reduced the diffusivity by more than 90% of the value corresponding to the agarose gel with no interpenetrating component. The effect of ALG was much less pronounced and rather inverse at first sight—the presence of ALG in the gels slightly increases the average diffusivity. Nevertheless, taking into account the confidence interval of the diffusivity values, no definite conclusions can be derived for an effect of ALG on the rate of R6G diffusion in the gels.

A similar difference in effects of the two interpenetrating polyelectrolytes was observed also for partition coefficients calculated using Equation (8). While the concentration of R6G in the gel at the boundary with the source solution was not significantly affected even by the highest concentration (0.01 wt.%) of ALG as compared to the reference agarose gel (see Table 2), the same content of PSS increased the partition drastically (to more than 10-fold higher average concentration compared to reference agarose gel). In a fact, partition coefficients determined in this study illustrate the dynamic partitioning of the solute during its diffusion into the gel. Equilibrium distribution of the solute between the solution and the gel could be described more accurately by the results of equilibrium absorption experiments such as those described in our previous study [55]. On the other hand, the diffusion experiments proposed here benefits mainly from providing the simultaneous monitoring of partitioning and diffusion parameters in the single experiment.

FCS analysis provides a complementary view on the transport properties of the gels as far as self-diffusion of individual, homogeneously distributed R6G molecules is monitored. Self-diffusion coefficient (*D_s_*) of the R6G molecules in a respective gel is determined here from the time evolution of correlation function that characterizes the time scales of fluctuations in the intensity of the R6G fluorescence. Different diffusion models can be used in the mathematical evaluation of the correlation function. In our work, we have applied the simplest model involving a single characteristic decay-time to the autocorrelation function providing one global diffusion coefficient of the present chromophores. The diffusion coefficient values determined for the semiIPN hydrogels are summarized in Table 1 and Table 2. It can be seen that for both polyelectrolyte components interpenetrating the agarose matrix, the self-diffusivity of R6G decreases with the concentration of the binding component in the gel, only the exact extent of the suppression of P6G diffusivity is different for PSS and ALG, respectively. In the case of PSS, the effect is slightly less pronounced than in the case of effective diffusivities determined by the macroscopic diffusion assay. On the other hand, a modest reduction of the rate of thermal motion of R6G is here found out also for ALG, unlike the macroscopic assay. The apparent discrepancy between the results of macroscopic and FCS diffusivity assays may be attributed to the different experimental conditions as well as to the distinct physical phenomena behind the two methods. First, the macroscopic diffusion assay uses the concentration of tracked diffusion probe (R6G) orders of magnitude higher than FCS. The relative content of freely moving RG6 molecules, not affected by the interpenetrated polyelectrolyte, must be significantly different in the two methods. Therefore, macroscopic diffusion assay may not be sufficiently sensitive to detect an effect of weakly binding components (such as ALG). On the other side, when the binding is strong enough to entirely immobilize the fluorescent molecule, this molecule becomes “invisible” for the FCS methods and the average self-diffusivity determined by FCS may be overvalued. Actually, in contrast to the macro-scale experiments with the UV-VIS absorption detection of diffusing R6G, the FCS technique can analyze only the motion of fluorescence-emitting molecules. Therefore, the strongly physically bound R6G molecules that lose the light-emitting ability via static fluorescence quenching are not monitored and do not contribute to the calculated diffusion coefficient any more. In general, results of the FCS diffusivity assay confirm that R6G molecules are subjected to an attractive interaction with both the polyelectrolyte components, whereby the interaction is significantly stronger in the case of PSS.

Aside from the determination of the self-diffusion coefficient of a solute, the FCS method can also provide some additional parameters which might be interpreted concerning the mode of binding of the solute by the polymer network. For instance, in the case of time-resolved FCS technique, the average diffusion coefficients of R6G in the analyzed volume are complemented with corresponding average fluorescence lifetimes. Table 1 and Table 2 show the values of the average fluorescence lifetime of R6G determined by TCSPC (time-correlated single photon counting) analysis of the time-resolved FCS experiment. Once again, a dissimilar effect has risen from the presence of ALG and PSS, respectively. The presence of PSS in the gel matrix leads to a slightly increased value of fluorescence lifetime, while higher content of ALG rather decreases the value. As far as the fluorescence lifetime is inversely proportional to rates of non-radiative de-excitation processes, its value is sensitive to a local environment in which a motion of the molecule occurs. In particular, the increase in fluorescence lifetime can be explained in terms of loss of the rotational freedom of the fluorophore caused by the R6G binding by PSS.

On the other hand, the R6G fluorescence lifetime, the value of which usually varies around 4 ns [63], is known to be highly concentration-dependent, as Förster energy transfer between monomers and weakly fluorescent stable dimers at higher R6G concentrations decreases the quantum yield and causes the fluorescence lifetime shortening [64]. Therefore, a decrease of fluorescence lifetimes in the gel with the highest ALG content may be assigned to a change in the spatial distribution of R6G molecules in the gel, which does not alter the rotational freedom significantly but causes colocalization of R6G molecules and the formation of R6G dimers. Similarly, concentration effects may explain also a difference in the average fluorescence lifetimes determined for agarose gels without polyelectrolyte components in the two independent experimental batches (compare the values for 0% ALG and PSS, in Table 1 and Table 2, respectively). As far as all the analyzed gels in the respective experimental batch were prepared simultaneously using the same R6G source solution and gelation conditions, it can be expected that the total R6G concentration in the gels is well comparable. On the other hand, in the case of a very low concentration of the R6G in the gel (order of nM), this is difficult to reproduce among the different experimental batches.

The qualitative difference in the effects of PSS and ALG on the fluorescence lifetime can be attributed to the dissimilar types of solute binding by the two polyelectrolytes. Electrostatic binding between opposite charges on the functional moieties of ALG and R6G molecules is less orientation-specific and, therefore, does not limit the rotation of the electrostatically bound R6G molecules significantly. On the other hand, this binding concentrates the fluorophore in the vicinity of oppositely charged polyelectrolyte which may enhance its aggregation. Unlike that, the presence of benzene moieties in the molecular structure of PSS complements the electrostatic attraction of R6G molecules with the planar stacking of the π electron-rich aromatic systems which results in significant loss of the rotational freedom of R6G and the corresponding increase in fluorescence lifetime.

### 3.3. Characterization of the Internal Structure of the Gels

Because both mechanical and transport properties of hydrogels are inevitably coupled with their internal structure, for a reasonable interpretation of results provided by the diffusion and rheological analyses of the studied hydrogels it is essential to provide their qualitative and, at best, quantitative structural characterization. For this purpose, we have included in our study also a complex structural assay comprising methods of either direct visualization or an indirect physicochemical mapping of the hydrogel network morphology.

**Figure 3:**
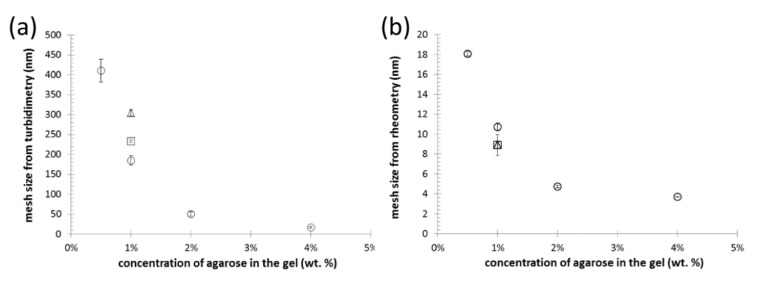
Results of indirect structural mapping of the hydrogels with different concentrations of agarose (**○**) and of the gels containing 1 wt.% of agarose-based interpenetrated by 0.01 wt.% of PSS (◻) and ALG (△), respectively. The analysis-specific effective mesh sizes were calculated from the results of turbidimetry (**a**) and (macro)rheometry (**b**), respectively.

For an indirect structural investigation of the agarose-based semiIPN hydrogels, we have at first utilized a simple turbidimetric assay, where turbidity is calculated from the optical density of the gels (determined by a standard transmission UV-VIS spectrometer) and plotted in log-log coordinates versus wavelength of the incident light. The exact mathematical apparatus for extracting internal structure parameters from such turbidity spectra was first proposed by Doty and Steiner [65] and further utilized by numerous authors [50,66,67,68,69]. We have followed the data-processing procedure that has been successfully applied by Aymard on various aqueous dispersions including swelled agarose hydrogels [50]. In this approach, linear regression of the log-log turbidity spectrum is performed in the range of wavelengths from 700 to 800 nm, and from the slope of the fitting line, the mesh size of an average scattering unit is determined (the log-log plots used for the calculation of the effective mesh size are shown in Figure S6 in Supplementary Materials). Aymard interprets the mesh size as the average distance between entanglements in the hydrogel network and, therefore, a value of this parameter can be taken as a rough estimate of the dimensions of pores in the gels. Mesh size values, determined from the turbidity data, are summarized in Figure 3a. As expected, the effective mesh size decreases significantly with the concentration of agarose in the gel as an indicator of the increasing density of crosslinking in the gel network. The order of magnitude of the calculated mesh sizes is in good agreement with the published range of pore sizes in agarose gels between 80 and 500 nm [70,71]. It was expected that the minor addition of polyelectrolyte interpenetrating components (PSS or ALG) will not remarkably alter the internal structure of the gels. It can be seen in Figure 3a that the addition of 0.01 wt.% of PSS or ALG increased in mesh size. The result can seem surprising as far as any addition of other polymer components should result in presence of more scattering centers which would hereby rather decrease the calculated mesh size value. Nevertheless, it must be emphasized that the method only characterizes a mean isometric dimension of the average scattering unit in the gel, and provides limited information about the other qualitative and quantitative structural parameters such as the actual shape of the network pores, width of the pore size distribution, etc. Therefore, conclusions about the results shown in Figure 3a should, rather, be about how the presence of minor contents of interpenetrating components does not significantly (in terms of the orders of magnitude) affect the size of the representative scatterer in the gel.

Not only the light scattering, but also the mechanical properties (mainly the elastic component of the deformation response) of the polymer networks are fundamentally connected with their crosslinking densities. Therefore, the results of rheometric assays may be used for the determination of effective mesh size, which in this case represents the mean size of the elastically active sections of polymer chains. Calculation of the mesh sizes from frequency sweep tests (using Equations (3) to (6)) is based on the rubber elasticity theory [72]. Although the validity of this theory for gels has continuously been questioned [73,74], it has been repeatedly utilized to calculate the mesh size of hydrogels based on rheometric parameters [52,75]. Previously, we have applied this approach in the determination of mesh size of polysaccharide-based phase-separated hydrogels [76]. The results of the calculations for the gels studied in the current study are shown in Figure 3b. As with the turbidimetry-derived values, also in this case the mesh sizes should be used for qualitative monitoring of changes in the internal structure rather than to provide absolute dimensions of the hydrogel pores. Again, it is evident that the increasing agarose content results in decreasing mesh size as a result of a more densely physically crosslinked agarose network. Compared to the turbidity-based mesh sizes, it can be seen that much lower values of the mesh size are calculated from the results of oscillatory rheometry. The elastically active chain sections differ naturally from those that participate in light scattering. Further comparing the results with published values of agarose gels pore sizes, it is also evident that the mesh size is significantly lower than the mean pore size. This is not a surprising fact when considering that also other than physical and chemical junction in the gel network (e.g., mechanical entanglements of the free polymer chains) can contribute to the elastic response to deformation. In this perspective, the most important outcome of this indirect structural mapping is that, in accordance with results from turbidimetry, no fundamental effect of the presence of interpenetrating polyelectrolyte component was observed (compare the effective mesh sizes for 1wt.% agarose gels with similar agarose gels containing also 0.01 wt.% ALG or PSS in Figure 3) as compared with the principal influence of the network-forming agarose component.

The gross picture of the morphology of the gels, provided by indirect structural mapping with rheometry and turbidimetry, was further refined by direct visualization of their internal porous structure by scanning electron microscopy (SEM). Cryogenic SEM (cryo-SEM) imaging was applied because aqueous samples cannot be directly observed in a high vacuum that needs to be maintained in the SEM chamber without any preceding stabilization. Therefore, the cryogenic fixation of the sample was performed via rapid cooling provided by the plunging of the sample in liquid nitrogen. To visualize the internal structure of a hydrogel sample, plunge freezing is followed by freeze-fracture (scratching the sample at high vacuum and cryogenic temperature) and freeze etching (letting the frozen water sublime to reveal the sample surface). Although plunging is not an optimal cryogenic fixation method for preparation of such hydrated samples in electron microscopy because of the Leidenfrost effect during which a thermally insulating film of vaporized nitrogen forms around the sample, preventing fast cooling and allowing water ice crystals to form inside the specimen [77,78], it could be also beneficial in the case of hydrogel structural studies. We assume that the size and distribution of the ice crystals correspond to the chemical composition of the hydrogels and the amount of free water.

**Figure 4:**
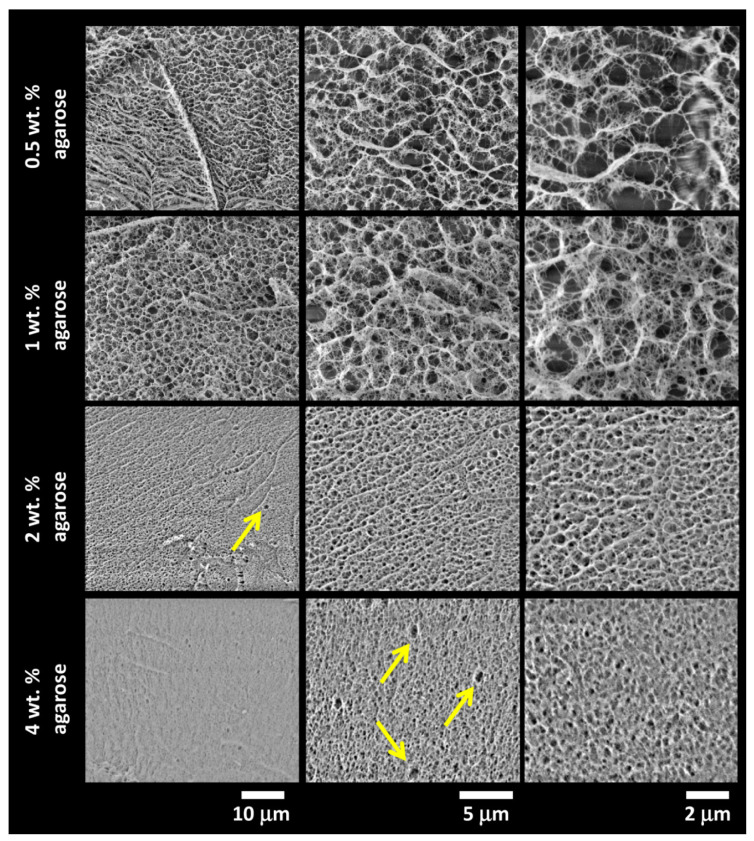
Results of cryogenic scanning electron microscopy (cryo-SEM) imaging of the internal structure of the plunge-frozen hydrogels with various content of agarose (with the absence of an interpenetrating polyelectrolyte component). Structure alterations caused by the formation of ice crystals are marked with arrows.

**Figure 5:**
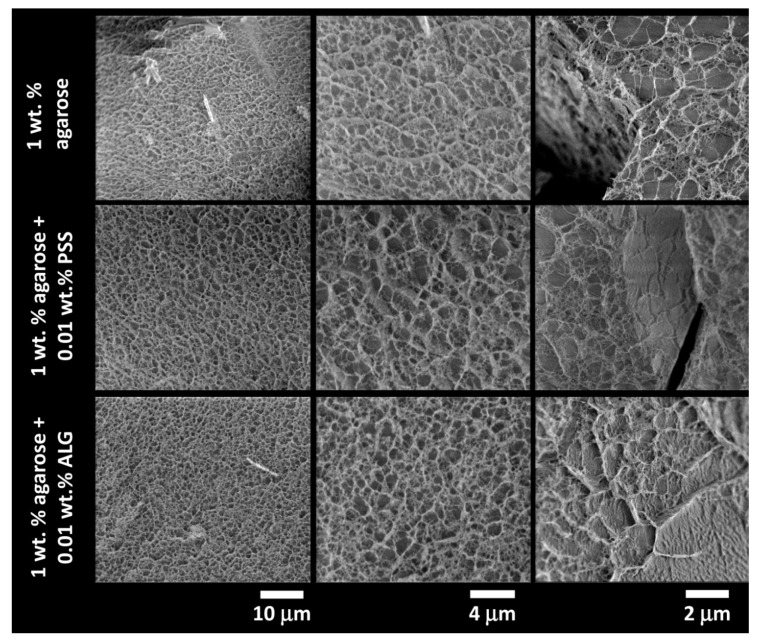
Results of cryo-SEM imaging of the internal structure of the plunge-frozen 1wt.% agarose hydrogels with and without the presence of an interpenetrating polyelectrolyte component (0.01 wt.% of PSS and ALG, respectively).

Results of the cryo-SEM imaging of the gels with different content of agarose (with the absence of an interpenetrating component) are shown in Figure 4, while the results for semiIPN hydrogels with the highest content of the respective interpenetrating component are provided in Figure 5. As expected, the principal role of agarose concentration in controlling the density of the crosslinking of the hydrogel network is obvious. On the other hand, as can be seen in Figure 5, even the highest applied concentration of an interpenetrating component (ALG or PSS) does not induce noticeable changes in the internal structure of the gels. Taking a closer look at the cryo-SEM images, it can be concluded that the plunge freezing of the gels in liquid nitrogen satisfactorily preserved the internal structure of the gels that is in correlation with the other applied methods. The network structure is largely isomorphic, with no apparent signs of anisotropic deformation during cooling. Nevertheless, some structural artifacts can be found in the images (such as those marked with arrows in Figure 4) indicating that the formation of ice crystals was not completely prevented.

The cryo-SEM images such as those shown in Figure 4 and Figure 5 serve primarily as a visual illustration of the qualitative characteristics of the internal structure of the studied gel networks. From this point of view, the visual evaluation of the cryo-SEM images confirms the qualitative findings provided by indirect structure-mapping methods (turbidimetry, rheometry), i.e., the principle that the network-forming role of agarose is not particularly disturbed by a presence of the polyelectrolyte component. Nevertheless, cryo-SEM imaging can also be further processed to support these qualitative conclusions from some quantitative outcomes. For this purpose, we have applied two techniques of the image processing that are implemented in the open-source scientific image-processing toolbox ImageJ and that are suggested for the analysis of porous structures.

**Figure 6:**
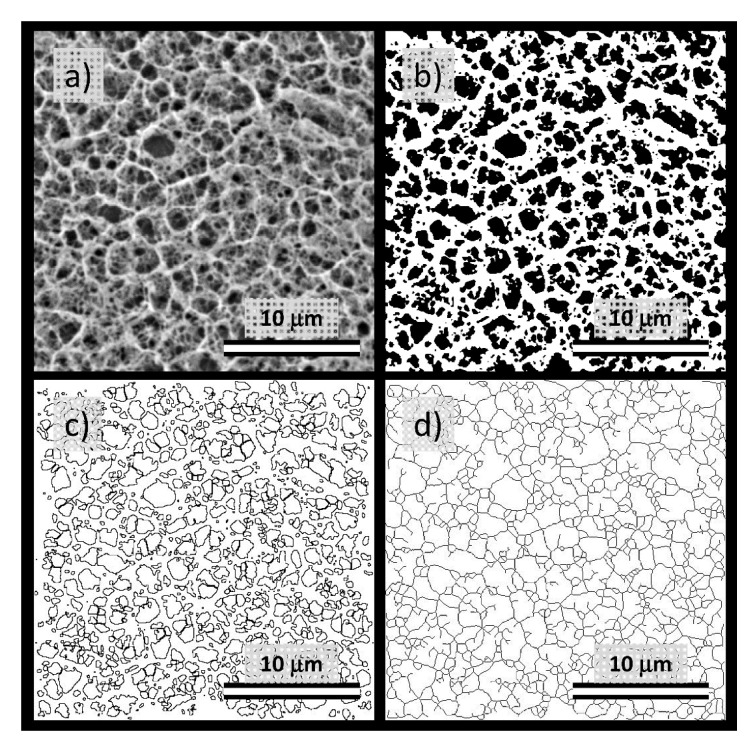
Processing of cryo-SEM images using the tools implemented in ImageJ software ((National Institute of Health, Bethesda, MD, USA and Laboratory for Optical and Computational Instrumentation, University of Wisconsin, Madison, WI, USA). Scalebar = 10 μm. (**a**) Original image (1 wt.% agarose gel without any interpenetrating component). (**b**) Binary projection (grayscale thresholding using MaxEntropy algorithm) of the original image. (**c**) An image mask is provided by the application of the ‘Analyze particles’ tool. (**d**) Image mask is provided by the application of the ‘Analyze skeleton’ tool.

**Figure 7:**
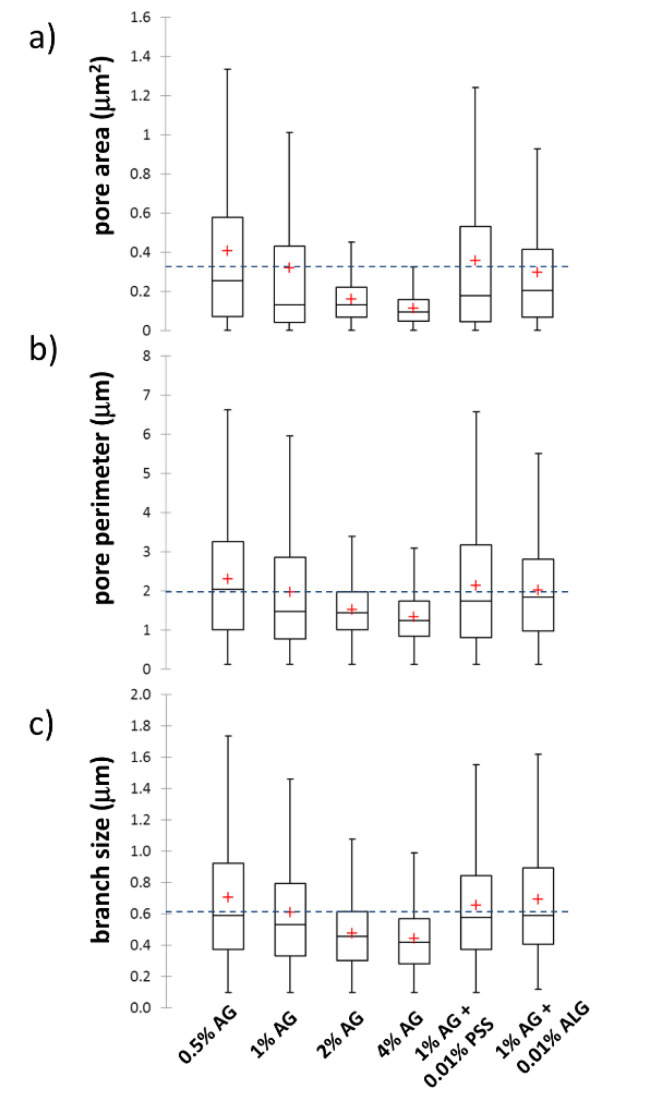
Box plots of pore size parameters determined from analysis of cryo-SEM images using ImageJ software. (**a**,**b**) Pore areas and perimeters determined using the ‘Analyze Particles’ tool. (**c**) Branch sizes using the ‘Analyze skeleton’ tool. Blue dashed lines in the box plots represent the mean values of the displayed parameters for 1 wt.% agarose gels without the presence of an interpenetrating polyelectrolyte component.

Firstly, the ‘Analyze particles’ tool (an automatic particle segmentation algorithm implemented in ImageJ) was used to identify individual pores in the image of the gel network. The outlines of the pores detected in the binary projection (Figure 6b) of an original image (Figure 6a) are shown in Figure 6c. As a numerical result of the Analyze particles tool, every outlined pore is described by its area and perimeter. Wherever it is necessary to take care of the pores which are displayed in the binary picture touching one another, the Watershed algorithm can be used before particle analysis. This algorithm uses a density profile to determine if one object with a peninsula should be two objects. If it determines that they should, it will draw a line to separate them. From the particle analysis, the distribution of pore areas and perimeters is obtained and processed into statistical parameters, e.g., average or mean values. The box plot projection of the pore areas and perimeters is shown in Figure 7a,b. These results again confirm that the size of pores, detected in the cryo-SEM images, decreases significantly with the increasing concentration of agarose in the gel. Once again, no such considerable pore size reduction is found for the semiIPN gels as a result of the presence of polyelectrolyte component.

Pore areas/perimeters are not in their absolute values reasonably comparable with the results of indirect structural analyses summarized in Figure 3. Furthermore, as can be seen in Figure 6c, the pores detected in the cryo-SEM images have complex, rarely isometric shapes and, therefore, neither pore areas nor pore perimeters can be simply transformed to an isometric representation of the pore size, such as the diameters in the case of circular pore projections. Therefore, aside from the ‘Analyse particles’ approach, we used also another image analysis option implemented in the ImageJ toolbox—the ‘Analyze skeleton’ tool. In this procedure, the network structure displayed in the analyzed image is first skeletonized, i.e., replaced by the line skeleton using a topology-maintaining medial axis thinning algorithm (example of the skeletonized representation of the processed image is shown in Figure 6d). Using the subsequent analysis tool, branches and junctions of such a skeleton are classified, counted, and measured. The Box plot which represents the statistical treatment of branch sizes, detected in the skeleton of cryo-SEM images of analyzed hydrogels, is shown in Figure 7c. Unlike the areas or perimeters of the pores, the mesh size represents a linear size parameter and as such can be directly compared with the effective mesh sizes determined by turbidimetry or rheometry. As can be seen in Figure 7c, the effect of agarose content in the gel surpasses the influence of the interpenetrating component also in the distribution of branch sizes. Increasing concentration of agarose induces denser crosslinking which results in shorter branches in the skeletonized image. The mean values of the branch sizes are in the order of hundreds of nm, which represents larger pore dimensions compared to the values determined by indirect structural techniques (Figure 3) as well as to the published pore sizes detected in agarose gels by other techniques [70,71]. This is probably caused by the partial expansion of dispersed water resulting from its imperfect vitrification and partial crystallization (see the signs of ice formation mentioned above).

## 4. Discussion

The work presented here was set two major objectives. The first was to evaluate a simple material strategy proposed for the development of hydrogel-based controlled release systems that would allow independent tuning of their mechanical and transport features. The strategy is based on hybrid (dual component) hydrogels that consist of a network formed by a hydrophilic gelling component with reduced affinity to bind the carried active substance, interpenetrated by chains of a linear polymer that possess functional groups prone to a strong attractive interaction with the active substance. The second objective was to propose and test an appropriate analytical methodology that could provide a complex insight into composition–structure–performance relationships in the resulting hydrogel materials with the main concern on mechanical and transport properties investigated not only on the macroscopic scale but also in the specific microcosms of the aqueous gel pores. Combining these two central viewpoints, the presented study represents a fundamental contribution to the state of the art in controlled release systems with the potential to open up a new area of research and development of hydrogel-based CR materials.

The idea of semiIPN hydrogels with rheological and transport properties tunable independently via manipulating the relative composition of a network-making and a binding component is amazingly simple and it is surprising that, to our best knowledge, no systematic effort has been made to evaluate the potential of the use of such materials in the development of novel CR systems. The approach has a great advantage in its modularity—a broad portfolio of gel-making hydrophilic polymers with a limited capacity of physical binding by strong interactions such as coulomb forces or hydrogen bonds can be found, including the materials most routinely used in research and development of the first generation hydrogels for drug-delivery systems (e.g., poly(vinyl alcohol) [79], poly(2-hydroxyethyl methacrylate) [3,80], poly(ethylene glycols) [81] or thermomelting polysaccharides like gellan, dextran or agar [82,83]). During their gelation process, all these matrices allow easy incorporation of the interpenetrating component—a linear hydrophilic polymer with the chemical structure selected concerning specific binding preferences of intended carried substance. In our study, charged polymers were suggested as the interpenetrating component as they are generally applicable as a binding component in CR systems carrying ionic active compounds. A wide range of polyelectrolytes bearing various densities of positive or negative charges in combination with diverse accompanying chemical functionalities may be found among natural polymers (anionic and cationic polysaccharides such as alginate, hyaluronan, chitosan, polypeptides poly-γ-glutamate, poly-lysine), their chemically modified analogs (trimethyl chitosan, quaternized dextran, etc.) or among fully synthetic polymers (e.g., polymerized substituted acrylic monomers like polyacrylates, polyacrylamides, substituted polystyrenes, etc.). As a prototype material, agarose-based hydrogels interpenetrated by a minor content (less than or equal to 1:100 ratio by weight) of anionic polyelectrolytes (PSS and ALG) were prepared and analyzed in this work. The structure, mechanical properties, and transport of positively charged organic solute (Rhodamine 6G) in these gels were described thoroughly.

**Figure 8:**
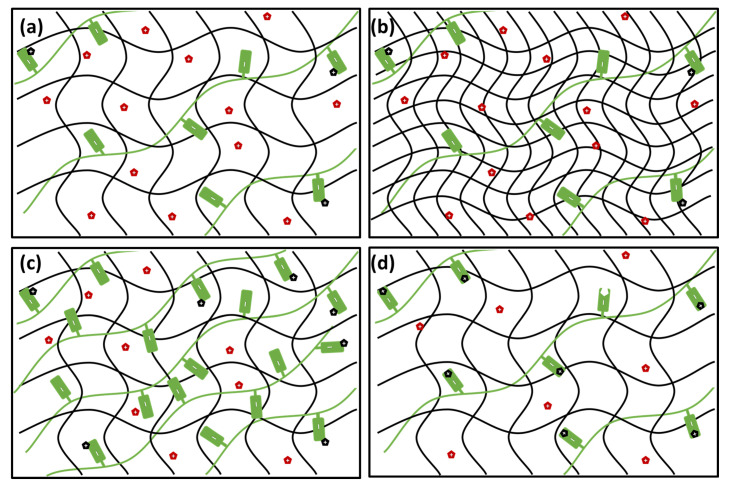
Schematic representation of the proposed concept of use of semiIPN hydrogels as controlled release (CR) systems with tunable rheological and transport properties. (**a**) Gelling component (black) with interpenetrating polyanion (green) possess universal binding affinity towards cations (red = free cations, black = bound cations). (**b**) With increasing content of the gelling component, gel stiffness and rigidity is increased with a negligible effect on the binding and diffusion of the cations. (**c**) With increasing content of the interpenetrating component, number of binding sites is increased affecting the transport properties of the gel with no effect on the mechanical properties. (**d**) Higher affinity of the binding sites (such as those provided for organic ions by PSS as compared with ALG) enhance the binding effectivity and shows more pronounced influence on the transport properties of the gel.

As expected, it was confirmed experimentally that the internal structure of the prepared gels in terms of their crosslinking density and corresponding internal porosity is principally governed by the concentration of agarose as the network-forming component (see the schematic representation of the effect in Figure 8a,b). As far as the morphology of the gel network predetermines its overall deformation response, also the mechanical performance of the gels is ruled by the content of agarose. By manipulating the concentration of the network-making component (agarose), the stiffness of the prepared gels may be altered over several orders of magnitude while the predominantly elastic (solid-like) rheological behavior typical for densely cross-linked hydrogels always prevails. Controlled stiffness of the hydrogel drug-delivery system is, on the one hand, a special concern mainly in load-bearing applications where the risk of premature mechanical destruction of a carrier is followed by a flow-away of the active compound which must be taken into account. On the other hand, a direct in vivo application of the highly elastic hydrogel materials may be problematic and limited to special physical forms such as gel micro- or nano-spheres [29]. Nevertheless, several strategies have been proposed to overcome this hydrogel-delivery issue. One of the most common approaches is based on the injection of a viscous polymer solution followed by in situ crosslinking induced by an appropriate environmental trigger. One example which may be fully compatible with the proposed strategy of a semiIPN-based hydrogel-controlled release system is represented by block copolymers with hydrophobic domains which can crosslink at increased (physiological) temperatures via reverse thermal gelation caused by an entropically driven aggregation of the hydrophobic blocks (e.g., triblock ABA copolymers of A = poly(ethylene oxide) (PEO) and B = poly(propylene oxide) (PPO) [84]). The controlled interpenetration of the networks prepared from these temperature-sensitive sol-gel systems with a linear binding polymer component should be free of experimental difficulties as far as several PEO-based semiIPN systems have already been studied [85,86]. From this perspective, the proposed hybrid-network concept was proved to provide the expected ability to manipulate easily the material viscoelasticity with existing options on how to meet the specific requirements on flow properties of materials used in contemporary CR applications.

Furthermore, it was confirmed by the systematic diffusion-mapping assay that transport of the carried compounds in the dual-component gels may be significantly influenced even by a trace content of a suitable interpenetrating component (again, the schematic representation of the effect is provided in Figure 8a,c). In the present work, we focused our attention on the molecular transport of Rhodamine 6G. This model solute was used on the one side for its specific molecular structure combining the positive charge on nitrogen atom with aromatic structural residues. This specific structural motif is common for numerous pharmaceutically active substances, e.g., for local anesthetics [47] or antibiotics [48]. Furthermore, well-described light absorption and emission behavior of R6G allows for the combination of various spectrometric techniques in the investigation of its transport in the hydrogel matrix on a different scale (macroscopic vs. microscopic scale). Results of experiments monitoring the diffusion of R6G from a source solution into the gels containing PSS confirmed that increasing the content of the interpenetrating component gradually suppresses the rate of R6G transport. In terms of the relative decrease in diffusion coefficient compared to a respective agarose gel, for the gels with ratio 1:100 (PSS to agarose) by weight, more than 90% decrease in diffusion coefficient was found for interpenetrated poly(styrene sulfonate). No such great alteration of R6G mobility may be achieved by manipulating the content of agarose itself, as was experimentally proved in our previous study [87]. Furthermore, not only the diffusion rate but also the partitioning of the solute in the gel is influenced significantly (more than a 10-fold increase in the partition coefficient was found in the case of PSS). Enhanced partitioning of the active compound may significantly increase the loading capacity of the drug carrier and consequently improve its pharmacokinetic profile [29]. On the other hand, a comparison of diffusion-related behavior of the gels interpenetrated by ALG and PSS, respectively, confirms that the degree to which the transport performance of the gel can be influenced is significantly dependent on the type of interaction between the active substance and the binding component (schematically represented in Figure 8a,d). In the case of ALG the binding of R6G is provided by the Coulomb electrostatic attraction alone while in the presence of PSS, we assume the stacking of aromatic moieties contributes to the binding significantly. This results in the fact that the extent to which the diffusion of R6G is affected (or, more precisely, the levels of R6G concentration at which these effects are manifested) differs significantly for the two interpenetrated polyelectrolytes.

Overall, on the example of prototype semiIPN hydrogels (in particular those containing PSS), it is illustrated that the transport properties of this type of hybrid network system may be altered greatly without any significant influence on their internal morphology and, hence, independently on their mechanical behavior. Furthermore, this strategy is not necessarily limited to the transport of hydrophilic active substances. Recently, several approaches have been proposed for the modification of hydrogels to provide controlled transport of water-insoluble solutes [88]. In our previous work, we have developed and characterized hydrogel systems with hydrophobic domains formed by surfactant micelles [89,90]. Controlled incorporation of such mechanically trapped hydrophobic domains in a supporting structure-defining hydrogel network could open new horizons in an independent tuning of mechanical and release behavior also for non-polar drug-releasing systems.

As was already noted, an additional merit of the present study is represented by the unique methodology that was utilized in providing the morphological, rheological, and also transport characterization of the studied hydrogels not only on the macroscopic but also on a microscopic scale. It was confirmed that this original analytical approach is highly beneficial in explaining the causal link between chemical composition, internal morphology, and mechanical and transport properties of the gels. For instance, our results clearly illustrate how the oscillatory (macro-)rheometry is complemented with the information provided by microrheometry in the completion of the overview of specific contributions of the polymer network and the surrounding aqueous solution, respectively, to the elastic and viscous components of the deformation response. Similarly, the involvement of a self-diffusion assay on the scale of individual molecules in parallel to common macroscopic monitoring of the diffusion of the same substance in the concentration gradient helps to explain how the binding of individual molecules in their local environment affects the rate of their molecular transport. Of course, the particular techniques described here do not represent the only and irreplaceable analytical option. For example, alternative techniques suggested for microrheological characterization of hydrogels include video microscopy [91] or fluorescence correlation spectroscopy [92]. Similarly, macroscopic diffusion experiments with hydrogels are often performed in a diffusion cell apparatus [87] while the self-diffusion of the solutes in the gels is commonly monitored via nuclear magnetic resonance (NMR) [93]. Nevertheless, a combination of the macroscopic and microscopic scale of the analysis in a single study is still rather scarce.

Finally, for a reasonable discussion of deformation or transport performance of hydrogel materials, it is always necessary to have at one’s disposal an analytical tool for mapping changes in the internal structure of the gel. Here, we have shown that cryo-SEM imaging of the gels provides detailed qualitative (and in combination with appropriate image processing techniques also quantitative) structural information. As noted, some indicators of alteration of the internal structure by the freezing artifacts can be found in our results. Nevertheless, this could be overcome e.g., by the utilization of a more appropriate cryofixation technique such as high-pressure freezing [94]. Aside from the direct visualization of the internal structure, our study also shows that valuable approximate structural information can be achieved also from much more accessible techniques of indirect structural mapping such as turbidimetry, rheometry, and additionally, for example, also differential scanning calorimetry [95].

## 5. Conclusions

It this study, we have experimentally verified through the results of a complex multiscale analysis of structure, viscoelastic, and transport properties of model agarose-based hydrogels with interpenetrating polyelectrolyte components that the concept of semiIPN gels containing an inert polymer network interpenetrated by a linear polymer component with the properly selected binding functionality can be successfully applied in the development of hydrogel materials with the ability of independent manipulation of mechanical and transport properties. Such materials possess a great application potential in controlled release systems, where one of the fundamental selection criteria for a suitable material candidate is represented by agreeing the required viscoelasticity and release-kinetic properties. In so far as this concept in general necessitates minimal requirements in the gelation procedure, it may be easily implemented in diverse state-of-the-art approaches in hydrogel preparation. Therefore, we believe that the presented work may become a stepping stone for a brand-new direction in the research and development of hydrogel-based controlled release systems. Furthermore, the original analytical approach designed and applied in this study is proposed as the methodological framework for these follow-up studies providing complex insights into composition–structure–performance relationships in developed hydrogel material.

## Figures and Tables

**Table 1:** Summarized results of investigation of R6G diffusion on the macroscopic and molecular scale in hydrogels with/without poly(styrene sulfonate) (PSS).

Weight Content of PSS in the Gel	Macroscopic Diffusivity *D_eff_* (µm^2^·s^–1^)	FCS Diffusivity * D_s_ (µm^2^·s^–1^)	Partition Coefficient ε (-)	Fluorescence Lifetime τ (ns)
0%	389 ± 7	343 ± 7	5.3 ± 2.0	4.21 ± 0.06
0.002%	158 ± 27	196 ± 27	10.2 ± 2.6	4.21 ± 0.09
0.005%	52 ± 25	148 ± 25	31.0 ± 10.1	4.27 ± 0.07
0.010%	27 ± 22	106 ± 22	64.8 ± 36.7	4.30 ± 0.06

* self-diffusion coefficients determined via fluorescence correlation spectroscopy.

**Table 2:** Summarized results of investigation of R6G diffusion on the macroscopic and molecular scale in hydrogels with/without alginate (ALG).

Weight Content of ALG in the Gel	Macroscopic Diffusivity *D_eff_* (µm^2^·s^–1^)	FCS Diffusivity *D_s_* (µm^2^·s^–1^)	Partition Coefficient ε (-)	Fluorescence Lifetime τ (ns)
0%	333 ± 33	385 ± 15	7.0 ± 1.6	3.70 ± 0.02
0.002%	379 ± 27	309 ± 21	6.4 ± 1.2	3.73 ± 0.03
0.005%	379 ± 46	304 ± 48	6.4 ± 1.0	3.69 ± 0.01
0.010%	394 ± 42	298 ± 21	6.1 ± 1.0	3.64 ± 0.01

## Supplementary Materials

The following are available online at https://www.mdpi.com/2073-4360/12/11/2561/s1, Figure S1: Results of frequency sweep rheometry tests for various compositions of agarose-based hydrogels presented as frequency dependencies of storage (a) and loss (b) moduli, respectively. Figure S2: Results of frequency sweep rheometry tests for hydrogels with different contents of interpenetrating components—PSS (a,b) and ALG (c,d)—represented by frequency dependencies of storage (a,c) and loss (c,d) moduli. Figure S3: Results of frequency sweep rheometry tests for hydrogels with different contents of interpenetrating components—PSS (a,b) and ALG (c,d)—represented by frequency dependencies of complex moduli (a,c) and phase angle (b,d). Figure S4: Results of strain sweep rheometry tests for various compositions of agarose-based hydrogels presented as frequency dependencies of storage (a), loss (b) and complex (c) moduli, and phase angle (d), respectively. Figure S5: Results of DLS microrheometry tests for hydrogels with different contents of interpenetrating components—PSS (a, b) and ALG (c,d)—represented by frequency dependencies of complex moduli (a,c) and phase angle (b,d). Figure S6: Results of turbidimetry presented as log-log plot of turbidity vs. wavelength (between 700 and 800 nm). Slope of the linear regression was used to calculate the effective mesh size according to Aymard [50].
