# Supplementary material for: Multiscale Experimental Evaluation of Agarose-Based Semi-Interpenetrating Polymer Network Hydrogels as Materials with Tunable Rheological and Transport Performance
Source: Polymers (Basel). 2020 Oct 31;12(11):2561. doi: 10.3390/polym12112561 (PMC7693122; doi:10.3390/polym12112561)
Supplement: Supplementary file 1 [file polymers-12-02561-s001.pdf]

Supplementary material to:

Multiscale experimental evaluation of agarose-based semi-interpenetrating polymer network hydrogels as materials with tunable rheological and transport performance

Monika Trudicova[1], Jiri Smilek[1], Michal Kalina[1], Marcela Smilkova[1], Katerina Adamkova[2], Kamila Hrubanova[2], Vladislav Krzyzanek[2] and Petr Sedlacek[1]*

[1] Faculty of Chemistry, Brno University of Technology, Purkynova 118, 612 00 Brno, Czech Republic.
[2] Institute of Scientific Instruments of the Czech Academy of Sciences, v.v.i., Kralovopolska 147, 612 64 Brno Czech Republic.

* Correspondence: sedlacek-p@fch.vut.cz; Tel.: +420 541 149 486

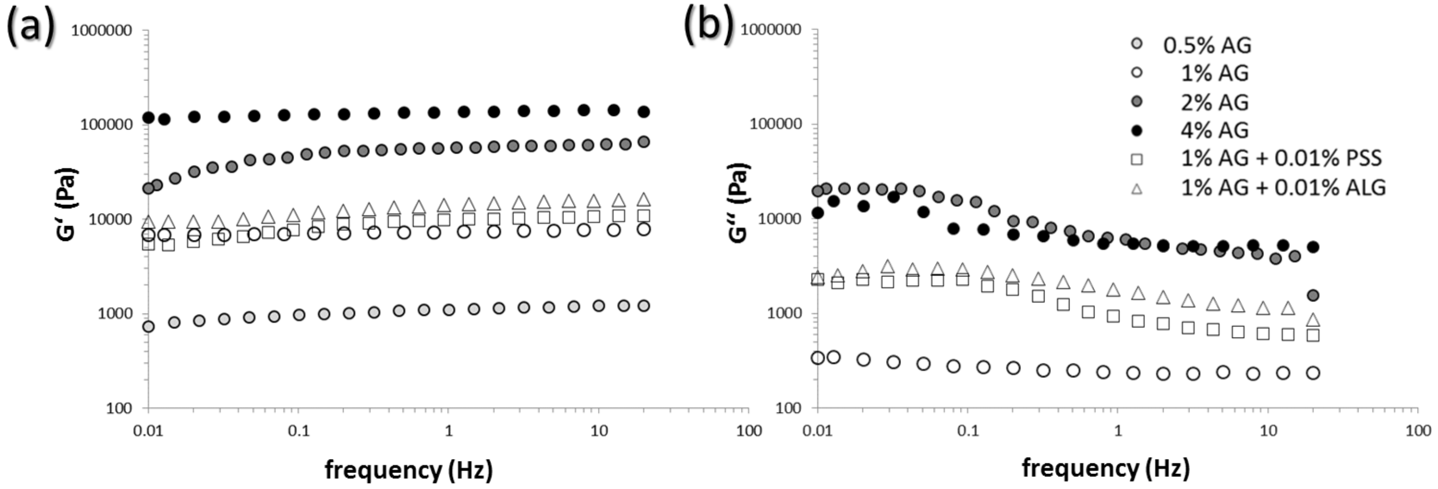

Figure S1 Results of frequency sweep rheometry tests for various compositions of agarose-based hydrogels presented as frequency dependencies of storage (a) and loss (b) moduli, respectively.

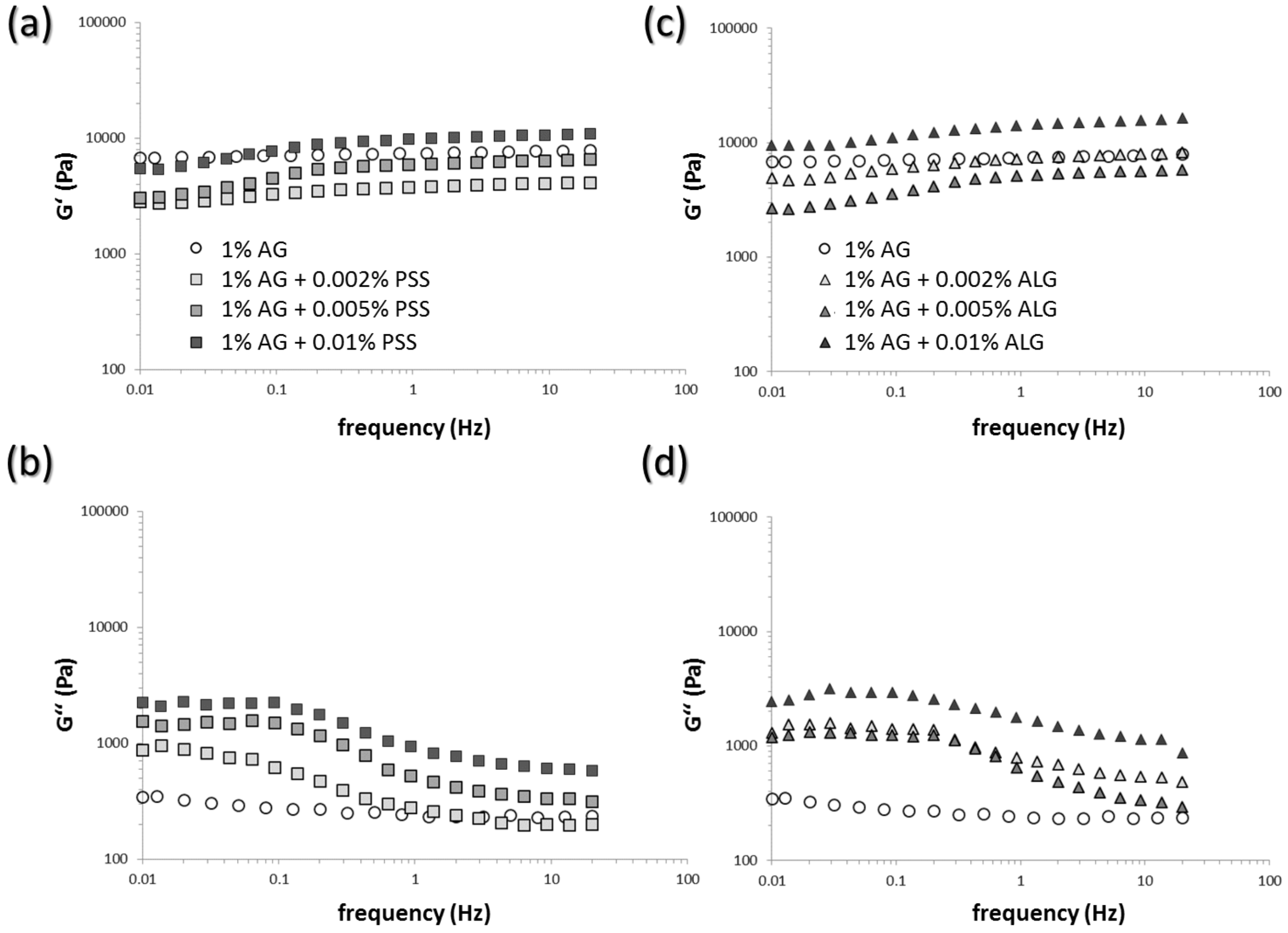

Figure S2 Results of frequency sweep rheometry tests for hydrogels with different contents of interpenetrating components – PSS (a, b) and ALG (c, d) – represented by frequency dependencies of storage (a, c) and loss (c, d) moduli.

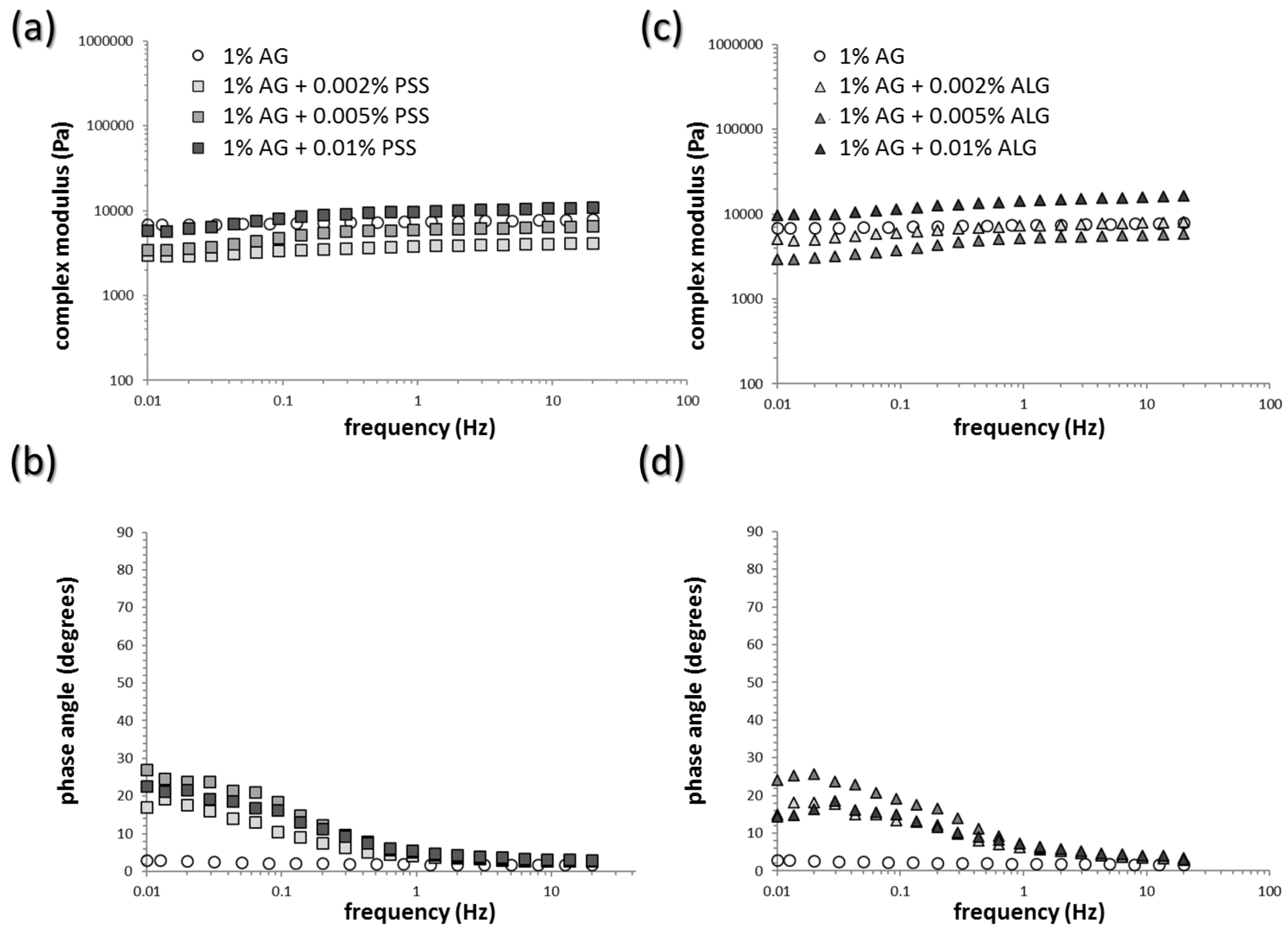

Figure S3 Results of frequency sweep rheometry tests for hydrogels with different contents of interpenetrating components – PSS (a, b) and ALG (c, d) – represented by frequency dependencies of complex moduli (a, c) and phase angle (b, d).

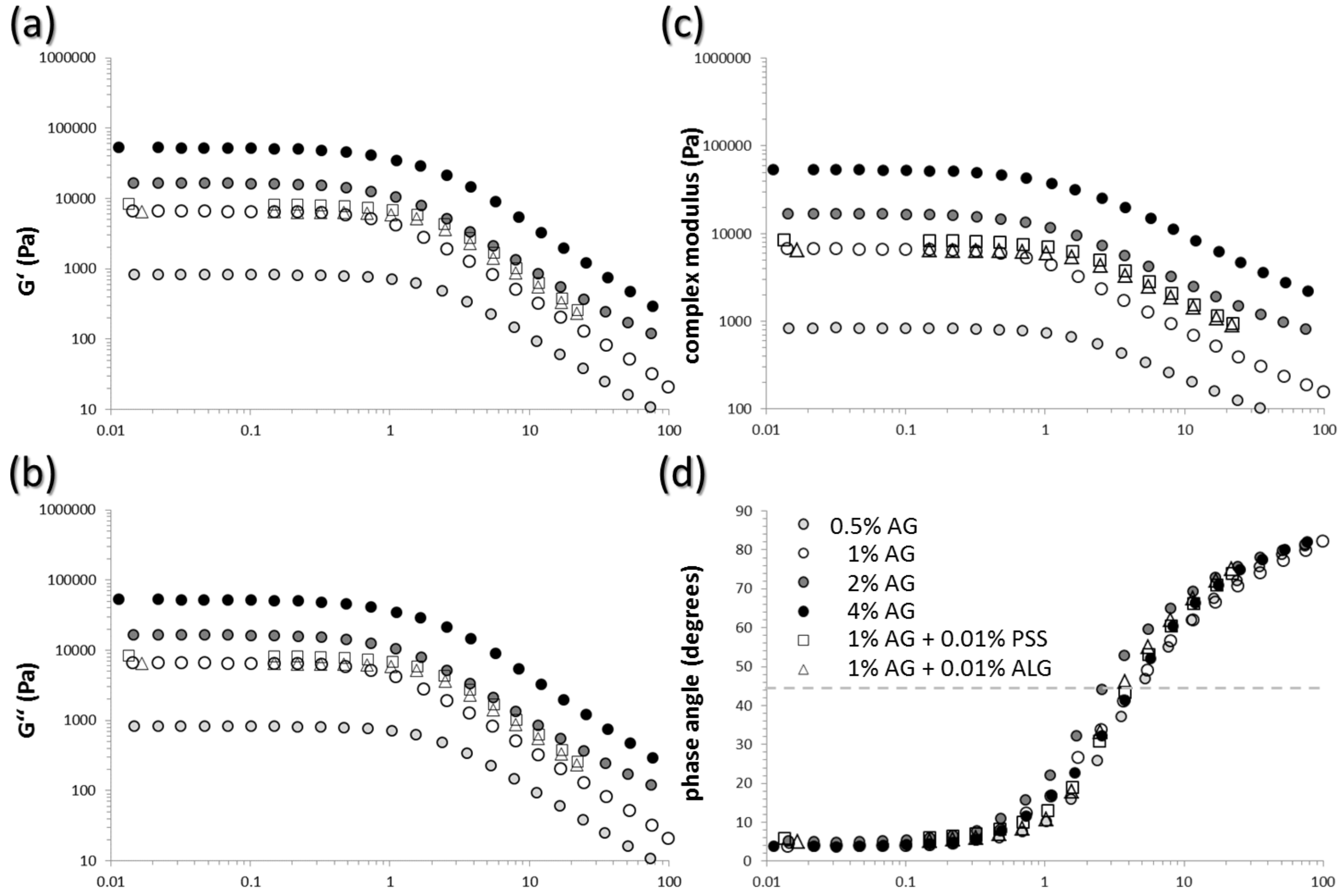

Figure S4 Results of strain sweep rheometry tests for various compositions of agarose-based hydrogels presented as frequency dependencies of storage (a), loss (b) and complex (c) moduli, and phase angle (d), respectively.

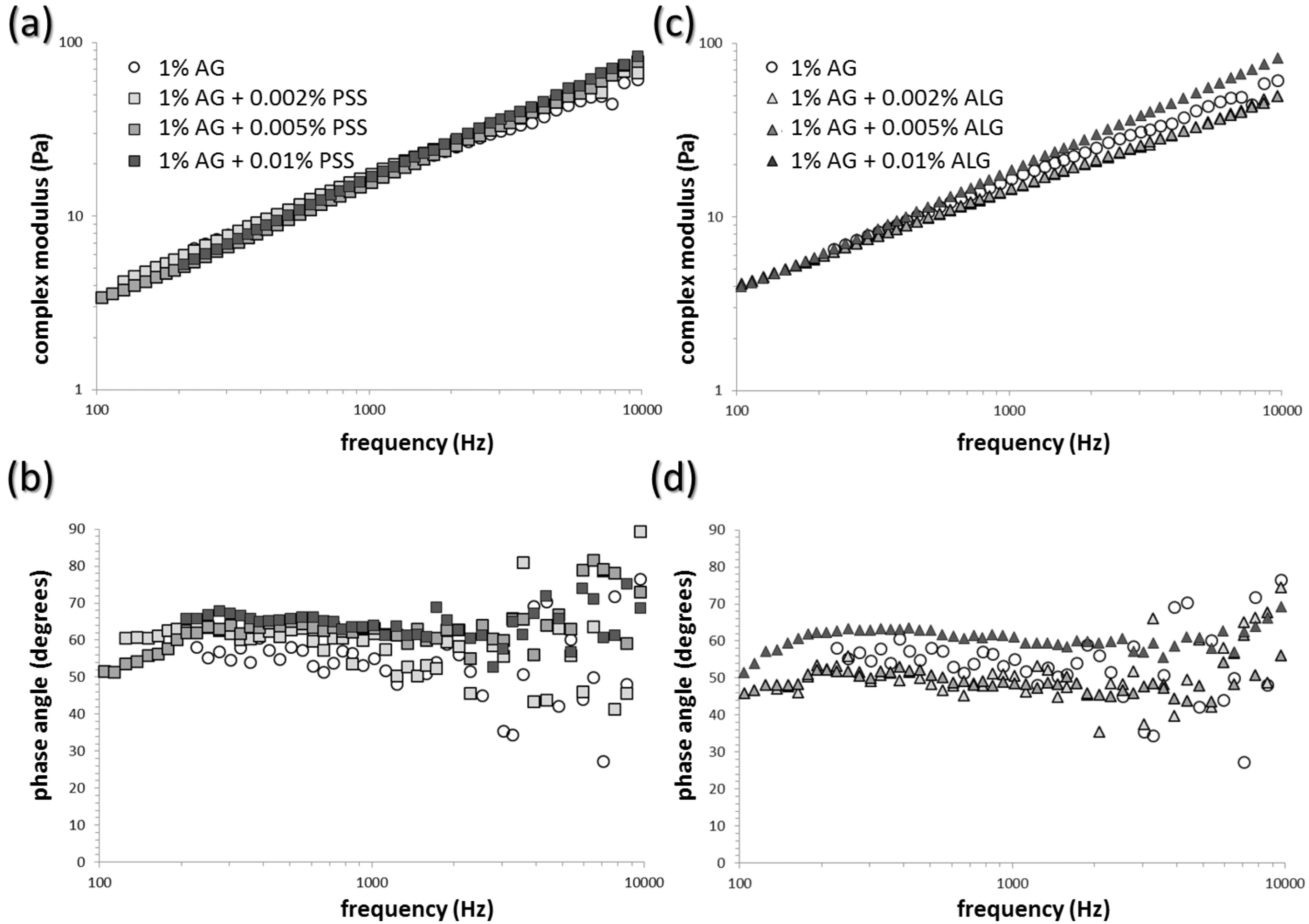

Figure S5 Results of DLS microrheometry tests for hydrogels with different contents of interpenetrating components – PSS (a, b) and ALG (c, d) – represented by frequency dependencies of complex moduli (a, c) and phase angle (b, d).

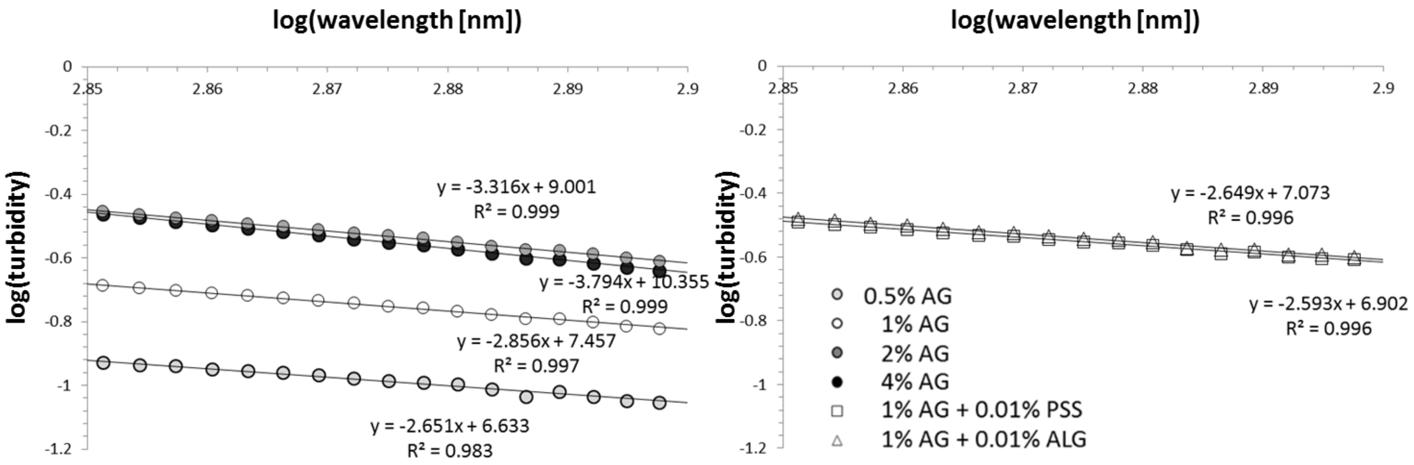

Figure S6 Results of turbidimetry presented as log-log plot of turbidity vs. wavelength (between 700 and 800 nm). Slope of the linear regression was used to calculate the effective mesh size according to Aymard [50].
